# Cost Per Participant Recruited From Rural and Remote Areas Into a Smoking Cessation Trial Via Online or Traditional Strategies: Observational Study

**DOI:** 10.2196/14911

**Published:** 2019-11-12

**Authors:** Judith Byaruhanga, Flora Tzelepis, Christine Paul, John Wiggers, Emma Byrnes, Christophe Lecathelinais

**Affiliations:** 1 University of Newcastle Callaghan Australia; 2 Hunter New England Population Health Wallsend Australia; 3 Hunter Medical Research Institute New Lambton Heights Australia

**Keywords:** smoking cessation, tobacco use, rural population

## Abstract

**Background:**

Rural and remote residents are more likely to smoke than those who live in major cities; however, recruitment of research participants from rural and remote areas can be challenging. The cost per participant recruited from rural and remote areas via online (eg, social media) and traditional strategies (eg, print) has implications for researchers on how to allocate resources to maximize the number of participants recruited. Participant characteristics such as demographics, financial stress, mental health, and smoking-related factors may be associated with recruitment method (ie, online vs traditional), and so it is important to understand whether certain subgroups are more likely to be recruited via a particular strategy.

**Objective:**

This study aimed to determine the cost per participant recruited and examine whether characteristics such as demographics, financial stress, mental health, and smoking-related factors may be associated with the recruitment method (ie, online vs traditional).

**Methods:**

Participants were recruited into a randomized trial that provided smoking cessation support. Eligible participants were aged 18 years or older; used tobacco daily; had access to video communication software, internet, and telephone; had an email address; and lived in a rural or remote area of New South Wales, Australia. This study describes the natural (observed) experience of recruiting participants via online and traditional methods into a smoking cessation trial.

**Results:**

Over 17 months, 655 participants were recruited into the smoking cessation trial. A total of 88.7% (581/655) of the participants were recruited via online methods. Moreover, 1.8% (12/655) of the participants were recruited from remote locations and none from very remote areas. The cost per participant recruited by the various online strategies ranged from Aus $7.29 (US $4.96, £4.09, and €4.43) for Gumtree, a local online classified website, to Aus $128.67 (US $87.63, £72.20, and €78.28) for email. The cost per participant recruited using traditional strategies ranged from Aus $0 (US $0, £0, and €0) for word of mouth to Aus $3990.84 (US $2757.67, £2227.85, and €2477.11) for telephone. Women had greater odds of being recruited via online methods than men (odds ratio 2.50, 95% CI 1.42-4.40). No other characteristics were associated with the recruitment method.

**Conclusions:**

The cost per participant recruited via online and traditional strategies varied, with the range being smaller for online than traditional recruitment strategies. Women have greater odds of being recruited via online strategies into rural smoking cessation trials.

**Trial Registration:**

Australian New Zealand Clinical Trials Registry ACTRN12617000514303; https://www.anzctr.org.au/Trial/Registration/TrialReview.aspx?id=372584&isReview=true

## Introduction

### Tobacco Use in Rural Population

In high-income countries such as Australia [1] and the United States [2], people who live in rural and remote areas have been identified as a priority population for smoking cessation interventions. Research has found that factors such as lower income [3], lower education levels [4], and fewer substance abuse treatment facilities [4,5] contribute to higher rates of tobacco use in rural areas, and rural residents are more likely to smoke tobacco than their counterparts who live in major cities [4]. Rural and remote locations comprise all areas outside major cities [1,4]. In the United States, the smoking prevalence is 24.9% in rural areas [6] compared with 20.8% of US adults in the general population [7]. In Australia, those in remote and very remote areas are 1.7 times more likely to smoke than those in major cities [8]. In Australia, the smoking rate is 12.8% [9] in the general population compared with 22% [10] among those living in regional and remote areas. There is, therefore, a need for tobacco control research to focus on such high-risk populations; however, recruitment of research participants who live in rural or remote locations for smoking cessation interventions can be challenging [11]. Several factors such as large geographical areas, limited resources, and transport barriers contribute to the difficulty of recruiting participants from these locations [12].

### Recruitment Methods

Timely recruitment of participants is a very important aspect of large research studies [13] because delays in recruitment may increase financial costs [14], reduce the sample size obtained, and limit the study’s robustness through inadequate statistical power [15,16], resulting in the reduced possibility of detecting a statistically significant result when there is a true difference between treatments [17]. Researchers conducting trials, therefore, often explore several avenues to recruit participants ranging from online to traditional methods. Traditional recruitment approaches that have been used include print (eg, direct mail, newspapers, posters, and flyers), broadcast advertising (eg, radio and television advertisements) [18], and word of mouth. Online methods such as social media (eg, Facebook and Twitter) [19,20], online advertisements (eg, Google advertisements), email, and other website promotions have also been used to recruit participants into research [21-23]. The potential reach of online recruitment strategies makes them appealing for recruiting participants into smoking cessation programs [23-25] and smoking cessation trials [26-29]. For example, in 2018, there were 3.9 billion internet users [30], and in 2016 to 2017, 86% of all households in Australia had access to the internet [31]. Specifically, 82.7% of those living in inner regional locations of Australia have internet access at home, as do 80.7% in outer regional locations and 77.1% in remote areas [31].

There is also evidence that online recruitment strategies are able to recruit participants with different demographic characteristics to those recruited via traditional recruitment methods [26-28,32,33]. Google advertisements, links on websites [27], and Facebook advertising are significantly more likely to recruit smokers aged between 18 and 25 years [32] or find a 7-year age difference between traditional and online recruitment [26]. In contrast, participants recruited by traditional methods such as newspapers, flyers, radio, and word of mouth in another smoking cessation trial were older and more confident in their quit attempts compared with those recruited via online methods [28]. Furthermore, people with depression have been found to be more likely to use the internet for health purposes [34,35], and social media users are more likely to have mental health conditions [36]. Financial stress, defined as “the difficulty that an individual or household may have in meeting basic financial commitments due to a shortage of money” [37], may also affect whether people have access to the internet and online advertisements; however, no research has examined whether financial stress is associated with recruitment strategy (ie, online vs traditional).

### Cost of Recruitment

Traditional and online recruitment strategies have been found to be cost effective [26,28,29,38]. For example, in 2016, an Australian study reported that the cost of an enrolled participant into a smoking cessation trial was Aus $56.34 (US $38.93, £31.43, and €34.95) for online (social media) and Aus $52.33 (US $36.13, £29.20, and €32.46) for traditional recruitment strategies [28]. In another Australian smoking cessation trial, the cost of online social media recruitment was Aus $42.34 (US $29.24, £23.63, and €26.27) per participant enrolled and Aus $21.52 (US $14.86, £12.01, and €13.35) per participant for traditional recruitment strategies [26]. Furthermore, a US study with young adult smokers used Facebook and spent up to US $10 (£8.08 and €8.98) in 2014 for each participant recruited into the smoking cessation trial [38]. Similarly, in a study conducted in the United States using other online strategies but not Facebook (ie, an online health risk assessment [HRA], online advertisements, traditional material [offline promotions], and quit-line screening), young adult smokers were recruited for US $41.35 (£33.41 and €37.10) for each enrolled smoker [29]. Online materials recruited the most smokers (online advertisements: n=1426; US $41.35) for the lowest cost in comparison with the other 3 recruitment strategies (HRA: n=397, US $630.85; quit-line screening: n=189, US $133.61; and offline materials: n=1341, US $56.23) [29]. This cost information has also been presented as a table in Multimedia Appendix 1. The existing research on the cost of recruiting smokers into research trials and characteristics of participants recruited by online versus traditional methods has focused on the general population [26-29] and suggests that online recruitment is a feasible method that can complement traditional recruitment strategies [26,28].

### Objectives

To our knowledge, no studies have compared the cost per participant recruited and subgroups of smokers from rural and remote locations recruited using online and traditional strategies into a smoking cessation trial. Therefore, this study described the different methods used for recruitment of smokers from rural and remote areas and aimed to (1) determine the cost per participant recruited for each recruitment strategy and (2) examine whether demographic characteristics, financial stress, mental health, and smoking-related characteristics of the participants were associated with the type of recruitment method (ie, online vs traditional).

## Methods

### Design

This study used the baseline survey of a smoking cessation trial that assessed how participants were recruited into the study (ie, via online or traditional strategies). Participants were recruited to take part in a 3-arm randomized trial of behavioral support for smoking cessation [39]. The 3 arms of the trial provided (1) real-time video counseling delivered via video communication software (eg, Skype (Microsoft Corporation) and FaceTime (Apple Inc), (2) telephone counseling, or (3) written self-help materials (control) [39]. The University of Newcastle Human Research Ethics Committee granted ethics approval. The trial is prospectively registered with the Australian New Zealand Clinical Trials Registry (ACTRN12617000514303).

### Participants

Participants were recruited over a 17-month period from May 25, 2017, to October 2, 2018. To be eligible for the trial, smokers needed to be aged 18 years or older; use tobacco daily; have access to a mode of video communication (eg, Skype and FaceTime), the internet, and a telephone; have a current email address; and reside in an inner or outer regional, remote, or very remote area of New South Wales (NSW) Australia. The classification of these areas was based on the Accessibility and Remoteness Index of Australia (ARIA+) [40]. The ARIA+ is a geographic accessibility index that reflects the ease or difficulty people face in accessing services in nonmetropolitan Australia based on residential postcode [40]. The different degrees of rurality are established on road distances needed to be traveled from the location to service centers of various population sizes. The resulting index of geographic accessibility classification is then used to categorize locations as inner regional (>0.2 to ≤2.4), outer regional (>2.4 to ≤5.92), remote (>5.92 to 10.53), and very remote (>10.53) areas [40].

### Recruitment

Both traditional and online advertising methods were used concurrently to recruit participants. The success of the recruitment approaches was monitored on an ongoing basis, and recruitment resources were allocated to each method based on participant numbers recruited by each method throughout the recruitment period.

### Online Recruitment Strategies

For all the online strategies, there were advertisements throughout the entire recruitment period of May 2017 to October 2018.

#### Facebook

All Facebook advertisements targeted only adults in rural and remote postcodes that were eligible for this study as classified by the ARIA+. A total of 41 paid advertisements were placed on Facebook as needed to boost the recruitment rate with a fixed daily budget limit of Aus $30 to Aus $100 (US $20.73-US $69.10). Unpaid Facebook promotions were posted on a daily basis to more than 1161 Facebook community boards.

#### Twitter

A total of 38 Twitter posts were made from the study twitter account and used to promote the research project using the hashtag quit smoking (#quitsmoking).

#### Emails to Businesses and Individuals

A total of 11,858 emails obtained from a business directory and partners in health services, such as local health districts, were sent out to corporate businesses, health organizations, mining companies, local area businesses, and trades people located in the eligible areas.

#### Gumtree

A total of 9 advertisements were placed on Gumtree, a local free online classified website, in postcodes that were eligible, and these remained on the website for the entire recruitment period.

#### Web Promotions and Internet Search

A study website was set up at the beginning of the study to promote the study and was active for the entire study period. The website was visible to anyone that searched online search engines for smoking cessation information and was cited on all print and online study advertising material.

### Traditional Recruitment Strategies

#### Newspaper

The newspaper recruitment strategy was used from August 2017 to September 2018 with a total of 51 different rural and remote newspapers. Two sizes of paid advertisements were used (92×63 mm or 100×72 mm) and were placed in the middle section of the newspaper. There were 44 free newspaper articles and 7 paid newspaper advertisements in different rural and remote newspapers. The free articles were written by journalists together with the research team to promote the research.

#### Posters

In total, 68 Men’s Sheds located in rural and remote NSW were telephoned in October 2017 and asked to display a poster promoting the study on their premises. Of these, 10 premises indicated that they were willing to do so, and a colored poster was distributed to them. In May 2017, posters were sent to 28 gyms, 21 Police-Citizens Youth clubs, 5 dental centers, 2 pharmacies, 2 hairdressers, 1 laundry shop, and 1 restaurant. Given the geographic diversity of locations, the study team did not visit these sites to verify if in fact the posters were displayed.

#### Flyers

A total of 2400 flyers were distributed. Flyers were printed in color on 1 side of an A4 paper and trifolded. A total of 19,00 flyers were placed into residential mailboxes in inner and outer regional locations of NSW in June, July, November, and December 2017, and 500 flyers were distributed to attendees at a rural conference in September 2018.

#### Radio

A total of 6 live radio interviews on different radio stations targeting rural and remote areas were conducted in July, August, September, and November 2017 and September 2018. The interviews promoted the smoking cessation trial, including information about the study and how to enroll into the study.

#### Telephone Calls

Telephone calls were made to 2465 households (2284 landlines and 181 mobiles) from an unlisted landline between June 2017 and September 2018. A single call was made to these households to determine if there was a potentially eligible participant and, if so, whether they would be interested in enrolling into the study.

#### Magazine

A single, colored advertisement, 136×61 mm in size, that promoted the study was placed in the middle section of a rural magazine in April 2017.

### Data Collection Procedures

All the advertising materials promoted the study website. Potential participants could click on (for online) or type (for traditional) the study website address to access information about the smoking cessation trial. The study website included a detailed information letter that described the research, outlined the importance of the study, informed who was eligible to participate, and contained a hyperlink that potential participants could select to complete the online screening survey. The screening survey computer software determined whether eligibility requirements were met, and if participants were eligible, they were immediately redirected to the online baseline survey. The baseline survey took about 10 to 15 min to complete.

### Measures

#### Recruitment Method

To determine whether participants were recruited via an online or traditional strategy, each participant was asked how they heard about the study during the baseline survey. The response options were as follows: Facebook advertisement or page, Twitter, Gumtree, Google advertisements, Web promotions (eg, study promoted on websites), Instagram, email, posters, flyers, postcards, magazine, newspaper, television, radio, word of mouth, telephone, or other (please specify).

#### Sociodemographic Characteristics

The baseline survey assessed sociodemographic characteristics including age, gender, country of birth, Aboriginal or Torres Strait Islander origin, education, marital status, and occupational status.

#### Geographical Location

The participant’s residential postcode collected during the baseline survey was used to determine location of residence (ie, inner regional, outer regional, remote, or very remote) according to the ARIA+ [40]. ARIA+ is a geographic accessibility index that reflects the ease or difficulty people face in accessing services. It is based on road distances needed to be traveled from the location to service centers of various population sizes from a point to the nearest urban centers and localities in 5 separate population ranges [40]. The classification comprises major cities, inner regional Australia, outer regional Australia, remote Australia, and very remote Australia [40].

#### Mental Health

Anxiety and depression were assessed using the 4-item Patient Health Questionnaire-4 (PHQ-4). The PHQ-4 is a reliable and valid measure of depression and anxiety in the general population [41]. The participants were asked, over the last 2 weeks, how often they had been bothered by the following: (1) feeling nervous, anxious, or on edge; (2) not being able to stop or control worrying, (3) little interest or pleasure in doing things, and (4) feeling down, depressed, or hopeless. The response options were as follows: not at all, several days, more than half the days, or nearly every day.

#### Financial Stress

Financial stress was assessed by asking if in the past 6 months, the participant did any of the following because of a shortage of money: (1) could not pay electricity, gas, or telephone bills on time; (2) could not pay the mortgage or rent on time; (3) pawned or sold something; (4) went without meals; (5) was unable to heat home; (6) asked for financial help from friends or family; and (7) asked for help from a welfare or community organization. The response options were yes or no [42].

#### Quitting Intentions

Participants were asked what best described their intentions regarding quitting. The response options were as follows: will quit in the next 30 days, will quit in the next 6 months, may quit in the future but not in the next 6 months, never expect to quit, and do not know.

#### Nicotine Dependence

The Heaviness of Smoking Index (HSI), which comprises 2 questions (time to first cigarette of day and number of cigarettes per day), was used to assess nicotine dependence [43]. The HSI was used rather than the Fagerstrom Test for Nicotine Dependence [44] because it has fewer items and it has been found to have acceptable reliability and validity [45].

#### Confidence to Quit

Confidence to quit was assessed by asking “if you decided to give up smoking altogether, how likely do you think you would be to succeed?” The response options were as follows: (1) very likely, (2) fairly likely, (3) fairly unlikely, (4) very unlikely, and (5) do not know.

### Statistical Analysis

Statistical analyses were completed using SAS software version 9.3 (SAS Institute Inc). Categorical measures were described using frequencies and percentages. For continuous measures, means, medians, and standard deviations were reported. To assess the relationship between variables of interest and recruitment strategy, a multiple logistic regression model was run, which included the recruitment strategy as the dependent outcome and the following as independent variables: sociodemographic characteristics, mental health measures, financial stress, and smoking-related variables. Odds ratios (ORs) and 95% CIs were reported. A criterion for statistical significance of *P*<.05 was used.

### Cost Analysis

All monetary costs were recorded directly from invoices as they were incurred. Costs associated with each recruitment strategy were tracked and summarized. The traditional recruitment costs included staff time for telephone calls; preparing materials; delivering radio interviews; liaising with newspaper journalists; dropping materials into mailboxes; printing and postage of materials; and costs for paid newspaper advertisements, magazine advertisements, and resources (eg, printing materials). The costs associated with the online strategies included staff time to send emails; posting advertisements on Facebook, Twitter, and Gumtree; setting up the study website, and the cost of paid Facebook advertisements. The cost per participant recruited was calculated by summing the total cost for each recruitment strategy and dividing it by the number of participants recruited via that strategy.

## Results

### Participant Characteristics

Between May 25, 2017, and October 2, 2018, 655 participants were enrolled in the study. Moreover, 77.4% (507/655) of the participants were female, 87.0% (570/655) were Australian born, and 40.3% (264/655) had completed a certificate or diploma from a Technical and Further Education campus, which provided vocational education in Australia. The average age of the participants was 43.7 years, 55.4% (363/655) participants were married or living in a de facto relationship, 62.6% (410/655) were employed, and 73.0% (477/655) lived in an inner regional area. The mean number of cigarettes smoked per day was 19.0 (SD 9.2), and the average minutes to first cigarette after waking up was 37.3 (SD 86.5) min. The mean age participants started smoking regularly was 16.6 (SD 4.5) years. Almost half of the participants (48.6%, 318/655) had made a quit attempt in the last 12 months, 45.0% (295/655) intended to quit in the next 30 days, and 53.0% (347/655) were confident that they were likely to quit (Table 1).

**Table 1:** Participants’ characteristics (N=655).

Demographic characteristics			Value
Age (years), mean (SD); median			43.7 (11.8); 43.0
**Gender, n (%)**			
	Female		507 (77.4)
	Male		148 (22.6)
**Country of birth, n (%)**			
	Australia		570 (87.0)
	Other		85 (13.0)
**Aboriginal or Torres Strait Islander, n (%)**			
	Yes		53 (8.1)
	No or do not know		602 (91.9)
**Education, n (%)**			
	Completed primary school		5 (0.8)
	Completed years 7-9		52 (7.9)
	Completed year 10		127 (19.4)
	Completed Higher Secondary Certificate or leaving year 12		52 (7.9)
	Technical and Further Education Certificate or diploma		264 (40.3)
	University degree or higher		146 (22.2)
	Other		9 (1.4)
**Marital status, n (%)**			
	Never married		147 (22.4)
	Married or de facto		363 (55.4)
	Married but separated		46 (7.0)
	Divorced		74 (11.3)
	Widowed		16 (2.4)
	Don’t know		9 (1.4)
**Location, n (%)**			
	Inner regional		477 (73.0)
	Outer regional		164 (25.1)
	Remote		12 (1.8)
**Employment, n (%)**			
	Full time or part time or casual		410 (62.6)
	Home duties		73 (11.2)
	Permanently unable to work or ill		56 (8.6)
	Retired		35 (5.3)
	Student (full or part time)		27 (4.1)
	Unemployed		40 (6.1)
	Other		14 (2.1)
**Smoking characteristics**			
	Cigarettes per day (n=620), mean (SD); median		19.0 (9.21); 20
	Time to first cigarette (n=632), mean (SD); median		37.3 (86.5); 15
	Age (years) started smoking regularly (n=649), mean (SD); median		16.6 (4.5); 16
	**Quit attempt in the past 12 months, n (%)**		
		Yes	318 (48.6)
		No	335 (51.2)
		Don’t know	1 (0.2)
	**Quitting intentions, n (%)**		
		Will quit in the next 30 days	295 (45.0)
		Will quit in the next 6 months	229 (35.0)
		Will not quit in the next 6 months or never expect to quit or do not know	131 (20.0)
	**Confidence to quit, n (%)**		
		Very or fairly likely to quit	347 (53.0)
		Very or fairly unlikely to quit	168 (25.7)
		Do not know	140 (21.4)

### Recruitment Strategies

Table 2 outlines the percentage of participants recruited by online and traditional methods. A total of 88.7% (581/655) of the participants were recruited via online strategies and 11.3% (74/655) of the participants were via traditional strategies. The majority (83.5%, 547/655) of the participants reported that they heard about the study through Facebook, whereas 7.2% (47/655) were recruited via newspaper.

### Cost per Smoker Recruited

The cost per participant recruited for each online and traditional recruitment strategy are described in Table 2. The cost for each participant recruited by the different online strategies ranged from Aus $7.29 for Gumtree to Aus $128.67 for email. In contrast, for the traditional recruitment strategies, the cost per participant recruited ranged from Aus $0 for word of mouth and television (initiated and completed by television station at no cost to the study) to Aus $3990.84 for telephone recruitment.

### Comparison of Participant Characteristics Recruited via Online Versus Traditional Strategies

Table 3 compares the characteristics of participants recruited via online or traditional methods. Women had greater odds of being recruited via online strategies than men (OR 2.50, 95% CI 1.42-4.40; *P*=.002). There were no significant associations between any other participant characteristics and type of recruitment method.

**Table 2:** Participant recruitment via recruitment methods.

Recruitment method			Participants, n (%)		Total cost for each strategy								Cost per participant recruited						
					Aus $		US $		£		€		Aus $		US $		£		€
**Online methods**																			
	Gumtree	5 (0.8)		36.43		24.81		20.44		22.16		7.29		4.96		4.09		4.43	
	Web promotions and internet search	10 (1.5)		437.56		298.00		245.55		266.22		43.76		29.80		24.56		26.62	
	Twitter	1 (0.2)		61.52		41.90		34.52		37.43		61.52		41.90		34.52		37.43	
	Facebook	547 (83.5)		33,738.52		22,977.28		18,932.47		20,526.24		61.68		42.01		34.61		37.53	
	Email	18 (2.7)		2315.98		1577.32		1299.56		1409.07		128.67		87.63		72.20		78.28	
**Traditional methods**																			
	Word of mouth	19 (2.9)		0		0		0		0		0		0		0		0	
	Television (initiated or completed by television station—no cost to study)	1 (0.2)		0		0		0		0		0		0		0		0	
	Newspaper	47 (7.2)		2363.38		1609.60		1326.27		1437.90		50.28		34.25		28.22		30.59	
	Radio (live interviews)	2 (0.3)		205.55		139.99		115.36		125.06		102.78		70.00		57.68		62.53	
	Magazine	2 (0.3)		170.81		116.33		95.86		103.92		85.41		51.96		47.93		58.17	
	Posters	1 (0.2)		566.65		385.92		318.01		344.76		566.65		385.92		318.01		344.76	
	Flyers	1 (0.2)		2546.29		1734.18		1429.38		1549.64		2546.29		1734.18		1429.38		1549.64	
	Telephone	1 (0.2)		3990.84		2757.67		2227.85		2477.11		3990.84		2757.67		2227.85		2477.11	

**Table 3:** Comparison of participants recruited via online and traditional strategies.

Variable and categories		Online strategies (n=581)	Traditional strategies (n=74)	Odds ratio (95% CI)	*P* value
**Gender, n (%)**					.002
	Female	460 (90.7)	47 (9.3)	2.50 (1.42-4.40)	
	Male	121 (81.8)	27 (18.2)	Reference	
Age (years) mean (SD)		43.72 (11.7)	43.23 (12.8)	1.01 (0.98-1.03)	.52
**Education,** **n (%)**					.39
	Year 10 or less	166 (89.7)	19 (10.3)	1.56 (0.75-3.26)	
	Higher Secondary Certificate or year 12 or Technical and Further Education	290 (90.1)	32 (9.9)	1.50 (0.79-2.83)	
	University or tertiary	124 (84.9)	22 (15.1)	Reference	
**Marital status, n (%)**					.09
	With partner	328 (90.4)	35 (9.6)	1.61 (0.94-2.77)	
	Without partner	253(86.6)	39 (13.4)	Reference	
**Employment,** **n (%)**					.21
	Employed full or casual or part time	366 (89.3)	44(10.7)	1.45 (0.81-2.59)	
	Not employed	215 (87.8)	30 (12.2)	Reference	
**Aboriginal or Torres Strait Islander, n (%)**					.14
	Yes	49 (92.5)	4 (7.6)	2.55 (0.73-8.87)	
	No	532 (88.4)	70 (11.6)	Reference	
**Australian born, n (%)**					.15
	No	79 (92.9)	6 (7.1)	1.95 (0.79-4.81)	
	Yes	502 (88.1)	68 (11.9)	Reference	
**Anxiety, n (%)**					.87
	No	295 (88.3)	39 (11.7)	1.05 (0.57-1.95)	
	Yes	286 (89.1)	35 (10.9)	Reference	
**Depression, n (%)**					.41
	No	348 (88.3)	46 (11.7)	0.76 (0.40-1.46)	
	Yes	233 (89.3)	28 (10.7)	Reference	
**Financial stress, n (%)**					.73
	0	268 (88.5)	35 (11.6)	0.71 (0.29-1.72)	
	1-3	241 (89.3)	29 (10.7)	0.82 (0.34-1.96)	
	4-7	72 (87.8)	10 (12.2)	Reference	
**Quitting intentions, n (%)**					.31
	Will quit in the next 30 days	267 (90.5)	28 (9.5)	1.68 (0.86-3.28)	
	Will quit in the next 6 months	203 (88.7)	26 (11.4)	1.41 (0.71-2.80)	
	Will not quit within the 6 months, never expect to quit, and do not know	111 (84.7)	20 (15.3)	Reference	
**Confidence to quit, n (%)**					.40
	Very or fairly likely	313 (90.2)	34 (9.8)	1.25 (0.74-2.11)	
	Very or fairly unlikely or do not know	268 (87.0)	40 (13.0)	Reference	
**Heaviness Smoking Index score, n (%)**					.57
	0-2: low addiction	143 (87.2)	21 (12.8)	0.60 (0.23-1.58)	
	3-4: moderate addiction	307 (88.0)	42 (12.0)	0.65 (0.27-1.58)	
	5-6: high addiction	79 (90.8)	8 (9.2)	Reference	

## Discussion

### Principal Findings

This Australian study found that both online and traditional recruitment strategies could facilitate the recruitment of smokers who lived in rural and remote areas into a smoking cessation trial. The online strategy had a high yield of smokers residing in rural and remote locations, similar to other studies conducted with the wider general population [26,29,32,38,46-50]. This is the first study to compare the cost per participant recruited from rural and remote locations using online and traditional strategies into a smoking cessation trial. The findings provide insight on the variability of cost per participant depending on the recruitment strategy used. The cost per participant recruited ranged from Aus $7.29 to Aus $128.67 for online strategies and Aus $0 to Aus $3990.84 for traditional strategies. Word of mouth was, by far, the most inexpensive method per participant recruited because the study incurred no cost from this recruitment strategy. However, word of mouth would not have been sufficient to recruit enough participants into the smoking cessation trial and, therefore, is not practical on its own, although it is an inexpensive strategy.

Facebook recruited the largest number of participants in this study compared with other online strategies and traditional recruitment methods (although this would have been influenced by the most money being spent on Facebook). However, Facebook was neither the most inexpensive online recruitment method in terms of cost per participant recruited nor was it cheaper than every traditional recruitment strategy. Nevertheless, to maximize the number of rural and remote participants recruited into smoking cessation trials, there needs to be a balance between the cost per participant recruited and the capacity (ie, total participants’ that can access each method). Specifically, there would be substantially fewer participants recruited into the study if only the most inexpensive methods (eg, word of mouth and Gumtree) were used, as these strategies did not recruit as large a number of participants as Facebook.

### Comparison With Prior Studies

The cost per participant for recruiting rural and remote smokers is variable and comparable with recruiting smokers in the general population [26,28,29,32,38,49,50], especially given that this study included all the costs such as staff time and printing of flyers unlike other studies [26,28,29,38,49]. For instance, in a US study of a Web-based smoking intervention [49], the total advertisement cost per randomized participant was US $40.51 (£33.33 and €36.32) for Facebook, US $34.71 (£28.56 and €31.12) for Google, and US $20.30 (£16.7 and €18.2) for traditional sources but excluded the costs for personnel. In another Australian study, the cost of online social media recruitment was Aus $42.34 (US $29.24, £23.63, and €26.27) per participant enrolled in a smoking cessation trial, whereas for the traditional recruitment strategies, it was Aus $21.52 (US $14.86, £12.01, and €13.35) per participant [26]. However, these costs did not include call back costs for after-hours’ time and materials sometimes mailed out to the newspaper participants [26]. In 2016, another Australian study [28] reported that the costs to recruit an eligible respondent into a smoking cessation trial via online (social media) methods was Aus $57.34 (US $38.93, £31.43, and €34.95) versus Aus $52.33 (US $36.13, £29.20, and €32.46) for the traditional methods. However, some costs such as printing and distribution of flyers were not included, and radio interviews were conducted free of charge [28]. Another smoking cessation trial [29] reported that online advertisements cost US $41.35 (£33.41 and €37.10), whereas traditional materials cost US $56.23 per enrolled participant. Finally, another study of young adult smokers using Facebook recruitment reported a cost of US $8.80 per eligible participant recruited into a cessation trial [38].

The results from this study showed that compared with men, women had greater odds of being recruited via online strategies. This is similar to a study by Stanczyk et al [50] where the internet yielded a higher proportion of female smokers, compared with other strategies. However other smoking cessation trials [26,28,51] have found no association between gender and method of recruitment. Nonetheless, it should be noted that the majority of men in this study were still recruited via online methods. Bearing in mind that most participants recruited via online strategies were recruited via Facebook, the finding that women had greater odds of being recruited via online strategies might be explained by evidence that women, unlike men, considered Facebook an integral part of their life to connect daily [52] and were more likely to seek support, either social (Facebook) or otherwise [53], but this was not the case for men and, particularly, men living in rural areas [54]. The participation of fewer men in smoking cessation research compared with women is not surprising. For instance, in a systematic review of proactive telephone counseling for smoking cessation [55], 17 out of the 21 studies reported a greater proportion of women than men participated [55]. Similarly, in a smoking cessation trial conducted in rural Kansas, United States, only 35% of the sample were men [56].

There were no significant associations found for other characteristics between participants recruited via online and traditional strategies in this study. In contrast, a study by Frandsen et al [28] reported a significant association between social media recruitment and younger participants compared with those recruited by traditional methods into smoking cessation support. Furthermore, although there is evidence that social media users are more likely to have mental health conditions [36], this study did not find any association between anxiety or depression and the recruitment method.

### Limitations

The study had a number of limitations. First, access to the internet was an eligibility criterion and might have excluded some smokers who would have been interested in behavioral support and had no internet connection. Second, most people were recruited from inner or outer regional areas, and the recruitment strategies were only able to reach a small proportion of smokers from remote areas and no one from very remote areas. This may not be surprising given that few newspapers and online notice boards or local classified websites specifically targeted very remote areas. In addition, the study targeted only 0.001% of the NSW population that lives in very remote areas [57]. Third, another limitation is that self-report was used as the measure of how participants were recruited into the study. Although there are online systems to track users after having clicked on an online advertisement (eg, via Facebook), people who heard about the study from traditional sources (eg, hardcopy newspaper) could not be tracked via such a system. Finally, this study recruited only adults living in rural and remote areas of Australia, and therefore, the findings might have limited generalizability to other countries, particularly low- and middle-income countries.

### Conclusions

The cost per participant recruited via online and traditional strategies varied with the range of costs being smaller for online than traditional recruitment strategies. Women have greater odds of being recruited via online strategies into rural smoking cessation trials.

## Appendix

Multimedia Appendix 1 Supplementary table of studies reporting cost per participant recruited in the general population via online and traditional recruitment strategies (summarises literature in Introduction).

## Abbreviations

ARIA+: Accessibility and Remoteness Index of Australia
HSI: Heaviness of Smoking Index
HRA: health risk assessment
NSW: New South Wales
OR: odds ratio
PHQ-4: Patient Health Questionnaire-4
