# Supplementary material for: Cost Per Participant Recruited From Rural and Remote Areas Into a Smoking Cessation Trial Via Online or Traditional Strategies: Observational Study
Source: J Med Internet Res. 2019 Nov 12;21(11):e14911. doi: 10.2196/14911 (PMC6880230; doi:10.2196/14911)
Supplement: Multimedia Appendix 1 [file jmir_v21i11e14911_app1.pdf]

Supplementary Files

Studies reporting cost per participant recruited in the general population via online and traditional recruitment strategies

Study author and Country	Cost per participant enrolled	
	Online (Social Media)	Traditional
Frandsen M, et al, [27] Australia	AU$42.34 (US$29.24, £23.63, €26.27)	AU 21.52 (US$14.86, £12.01, €13.35)
Frandsen M et al, [29] Australia	AU 57.34 (US$38.93,£31.43, €34.95)	AU$52.33 (US$36.13, £29.20, €32.46)
Buller DB et al, [30] USA	US $41.35 (£33.41, €37.10)	US$ 630.85 (AU$933.06, £519.57, €563.13) (HRA)<br>133.61 (£110.03, €119.27) (Quitline)<br>$56.23 (£46.26, €50.41) (Offline material)
Ramo DE et al, [39] USA	US $10 (£8.22, €8.96)	Not applicable
